# The stigma perceived by people bereaved by suicide and other sudden deaths: A cross-sectional UK study of 3432 bereaved adults

**DOI:** 10.1016/j.jpsychores.2016.05.009

**Published:** 2016-08

**Authors:** Alexandra L. Pitman, David P.J. Osborn, Khadija Rantell, Michael B. King

**Affiliations:** a UCL Division of Psychiatry, University College London, Gower St, London WC1E 6BT, United Kingdom; b Camden and Islington NHS Foundation Trust, St Pancras Hospital, 4 Saint Pancras Way, London NW1 0PE, United Kingdom; c Education Unit, UCL Institute of Neurology, United Kingdom

**Keywords:** Bereavement, Guilt, Shame, Stigma, Suicide, Responsibility

## Abstract

**Objective:**

To test the hypothesis that perceived stigma scores in young adults bereaved by suicide are significantly higher than in young adults bereaved by other sudden deaths, whether blood-related to the deceased or not.

**Methods:**

We conducted a cross-sectional study of all staff and students aged 18–40 at 37 UK higher educational institutions in 2010, and identified 3432 respondents who had experienced a sudden bereavement of a close contact since reaching the age of 10, either due to sudden natural causes, sudden unnatural causes, or suicide. We used multivariable regression to compare scores on the stigma, shame, responsibility and guilt subscales of the Grief Experience Questionnaire, adjusting for socio-demographic factors and pre-bereavement psychopathology.

**Results:**

People bereaved by suicide (n = 614) had higher stigma scores than people bereaved by sudden natural death (n = 2106; adjusted coefficient = 2.52; 95% CI = 2.13–2.90; *p* = < 0.001) and people bereaved by sudden unnatural death (n = 712; adjusted coefficient = 1.69; 95% CI = 1.25–2.13; *p* = < 0.001). Shame, responsibility and guilt scores were also significantly higher in people bereaved by suicide, whether compared with bereavement by sudden natural death or sudden unnatural death. Associations were not modified by whether the bereaved was blood-related to the deceased or not.

**Conclusions:**

Stigma was perceived more acutely by the relatives and friends of those who died by suicide than those bereaved by other causes of sudden natural or sudden unnatural death. Their high levels of perceived stigma, shame, responsibility and guilt require qualitative investigation to identify whether these grief dimensions limit social functioning, help-seeking behaviour and/or support offered.

## Highlights

• No studies have confirmed if suicide confers more stigma than other sudden losses.
• We compared people bereaved by suicide, sudden natural causes, and sudden unnatural causes.
• We found suicide bereavement to be most stigmatising of sudden bereavements.
• Suicide bereavement was also associated with shame, guilt, and responsibility.
• Associations applied to both relatives and non-relatives.

## 1 Introduction

Supporting people bereaved by suicide is a key objective of many international suicide prevention strategies [1], [2], and this group are now known to have an increased risk of suicide, depression, and psychiatric admission compared with people bereaved by other mortality causes [3]. UK evidence shows that the risk of suicide attempt applies whether blood-related to the deceased or not [4], indicating that explanations for these adverse outcomes lie beyond familial factors. Suggestions include assortative relating, shared social adversity, stigma, and social modelling [3]. Identifying explanatory factors is a key step in designing suicide prevention interventions targeted at people bereaved by suicide, but as yet our understanding of these mechanisms remains theoretical. Stigma is of interest because it is distressing, influences help-seeking, limits support available, is linked to risk factors for suicidality (such as social isolation and hopelessness [5]), and may be more modifiable than other explanatory factors [6]. There is tentative evidence for its contribution to explaining adverse outcomes in people bereaved by suicide [4]. However, stigmatising attitudes are not unique to suicide, and may also be directed at people bereaved by accidental deaths [7] or preventable natural causes [8] for their links with someone judged to have exhibited risky health behaviour. To begin to understand the role of stigma after sudden death, we need to start by confirming whether the degree of stigma associated with suicide bereavement exceeds that associated with other losses [3].

Stigma is a term more commonly applied to characteristics such as psychiatric [5] or neurological illnesses [9], but is also well-described in relation to people who have experienced bereavement, particularly after suicide [6], [7], [8] and other unnatural losses [7]. Dimensions of stigma include public or personal stigma, perceived stigma, and self-stigma. Public and personal stigma are forms of enacted (or objective) stigma, manifested in mistrust, fear, negative bias, and stereotyping of the bereaved, as well as social embarrassment and avoidance [10]. Public stigma towards people bereaved by suicide originated in the Middle Ages, when legal, religious, and social sanctions against suicide arose as a deterrent within Roman Catholic, Jewish and Islamic communities [10], [11]. Such sanctions persist in the tendency of life insurance companies to refuse policies for families with a history of suicide, or delay pay-outs after suicide. Personal stigma is apparent in attitudes towards suicide as a failure of problem-solving, blaming both the deceased and their friends and family [10]. US studies of non-bereaved subjects show a greater tendency to ascribe blame to a person bereaved by suicide than one bereaved by accidental death, homicide, or natural death [12], and to avoid the bereaved for fear of the social rules governing such interactions [13]. Perceived stigma describes the awareness of others' stigmatising attitudes, and is a form of felt (or subjective) stigma [14]. For example, US parents bereaved by a child's suicide reported hurtful responses from family and friends after their loss [15]. When perceived stigma is internalised as self-stigma, it engenders feelings of shame and worthlessness [5], [9]. Whilst perceived and self-stigma can reduce help-seeking and awareness of support available, public and personal stigma can reduce others' willingness to offer support [10].

The stigma associated with suicide and other deaths has been documented extensively in the qualitative literature [7], [10], [16], but is less well described quantitatively [3]. Surveys have demonstrated higher stigma and shame scores in people bereaved by suicide when compared with people bereaved by natural mortality causes [3]. Direct comparisons with people bereaved by non-suicide unnatural causes, however, show that people bereaved by suicide report higher shame scores but no differences on stigma [3]. This would appear to suggest that feeling highly stigmatised applies to all those bereaved by unnatural causes (perhaps due to others' distaste over the nature of the loss), and that shame characterises suicide bereavement specifically. However, methodological problems, such as small sample sizes, unvalidated scales, and unadjusted analyses, render these findings inconclusive [3], [15].

Our objective was to determine, in a UK sample, whether people bereaved by suicide have a higher risk of suicide attempt and feel more stigmatised than those bereaved by other sudden mortality causes. We chose to focus on young adult, given concerns about their vulnerability to social modelling of suicidal behaviour [17]. Our study was primarily designed to test the hypothesis that young adults bereaved by suicide report higher rates of incident suicidal thoughts and attempts than young adults bereaved by other causes of sudden death. Our findings supporting this hypothesis are reported separately [4]. The current paper reports on the testing of our additional hypothesis that significant differences exist between stigma scores for people bereaved by suicide, sudden unnatural death, and sudden natural death. We predicted that stigma scores would be highest in people bereaved by suicide, lowest in people bereaved by sudden natural causes, and intermediate to the two in those bereaved by sudden unnatural causes. We predicted that the same patterns would apply to three other components of grief: shame, responsibility, and guilt. We also examined whether the predicted associations would apply whether the bereaved were blood-related to the deceased or not.

## 2 Methods

### 2.1 Study design and participants

We invited all young adults working or studying at UK higher education institutions (HEIs) to participate in a closed online cross-sectional survey about sudden bereavement: the UCL Bereavement Study. We considered this sampling frame to provide the most efficient, comprehensive and pragmatic means of recruiting a hard-to-reach population of young adults [18], whilst simultaneously minimising traditional biases associated with recruiting a help-seeking sample. All 164 HEIs in the UK in 2010 were invited to participate, following-up non-responding HEIs to encourage broad socio-economic and geographic representation. Over 20% of HEIs (37/164) agreed to take part, with a higher response (40%) from those classified as the more prestigious Russell Group universities. This provided an estimated sampling frame of 659,572 staff and students. The majority of participating HEIs agreed to send an individual email invitation with embedded survey link to each staff and student member, as per study protocol. For reasons of sensitivity ten HEIs modified this strategy, for example by emailing students only, using their weekly news digest email, or advertising via staff and student intranet. All recipients (whether bereaved or not) were invited to take part in a survey of “the impact of sudden bereavement on young adults”, with the aim of masking them to the specific study hypotheses. There was no accurate way of measuring response, as the denominator of bereaved people was not ascertainable using routine data or survey methods.

Inclusion criteria were as follows: people aged 18–40 who, after ten years of age, had experienced sudden bereavement of a close friend or relative. Early childhood bereavements were excluded to minimise recall bias, and to capture adult cognitive processing of negative life events. Sudden bereavement was operationalised as “a death that could not have been predicted at that time and which occurred suddenly or within a matter of days”. Exposure status was sub-classified, via self-report, as: bereavement by suicide, bereavement by sudden natural causes (e.g. cardiac arrest), and bereavement by sudden unnatural causes (e.g. road deaths). For respondents who had experienced more than one mode of sudden bereavement, we adopted a hierarchical approach: all those bereaved by suicide were classified as such, regardless of other bereavements. Those bereaved by non-suicide deaths were classified according to the person they had felt closest to. We based our sample size calculation on the primary outcome for our main study; suicide attempt. We estimated that a minimum of 466 participants would be required in any one group (two-tailed analysis; 90% power) to detect a doubling of the UK community prevalence of lifetime suicide attempt (6.5%) in young adult samples [19].

The study was approved by the UCL Research Ethics Committee in 2010 (ref: 1975/002). All participants provided online informed consent.

### 2.2 Procedures

Our online questionnaire (see Supplementary material) was designed in consultation with a group of young bereaved adults and bereavement counsellors, and piloted with individuals accessing support from four national bereavement support organisations in the UK. The questionnaire elicited quantitative data on socio-demographic and clinical characteristics, including eight putative confounding variables identified from existing literature and clinical judgement: age, gender, socio-economic status (using the UK Office for National Statistics Standard Occupational Classification [20]), pre-bereavement depression, pre-bereavement suicidal self-harm, pre-bereavement non-suicidal self-harm, other family history of suicide (excluding index bereavement), years since bereavement, and kinship to the deceased.

Our primary outcome was perceived stigma using the 10-item stigmatization subscale of the Grief Experience Questionnaire (GEQ) [21]. The GEQ is a standardised, validated, self-administered instrument for the assessment of the phenomenology of grief. It was developed following qualitative interviews with US individuals bereaved by natural causes, accidental death, and suicide [22]. The original scale was further validated and refined using factor analysis of responses from a sample of Canadian adults bereaved by natural causes, accidental death, and suicide [21]. The resultant eight subscales are: abandonment/rejection, stigmatization, search for explanation, guilt, somatic reactions, responsibility, self-destructive orientation, and shame/embarrassment. Responses to items in each subscale are rated using a 5-point Likert-style frequency scale, to generate a subscale score of 5 to 25. As in previous studies comparing the impact of different modes of bereavement, we compared GEQ subscales rather than overall GEQ score to delineate specific components of grief [23], [24], [25]. The stigmatization subscale (Box 1) captures perceived rather than personal stigma, and includes items describing discrimination and loss of social support [21].

As secondary measures we selected three related GEQ subscales: shame (capturing feelings of embarrassment about the circumstances of the death), responsibility (a perception of having caused the death), and guilt (a distressing perception of having done something wrong through action or inaction). Again, as in previous studies using the GEQ [26], we slightly modified the wording of the original stem to elicit reactions following the death of any close contact rather than specifically a spouse.

### 2.3 Statistical analysis

We summarised numerical variables using mean and standard deviation, or median and range depending on their distribution. We summarised categorical variables using count and percentages. We used the chi-square test and one-way analysis of variance to test for simple associations between confounding variables and outcome(s).

We categorised responsibility and guilt scores into tertiles (low/medium/high scores) because their skewed distribution violated assumptions required for linear regression (i.e. residuals are normally distributed), even when transformed to log values. Comparing tertile measures also provided easily interpretable estimates, without too much loss of information as a result of categorisation of the variables. Score category cut-off points were determined based each variable's data distribution, and were consistent with clinical scenarios. Thresholds for responsibility scores were low = ≤ 5; medium = 6–8; high = 9–25, and for guilt were low = 5–11.6; medium = 12.5–15.8; high = 16.6–25.

We used multivariable linear regression to model continuous outcomes (stigma and shame) using *xtmixed* commands in Stata [27], with HEI as random effect. We checked model assumptions using residual plots. We compared mean scores rather than using the standardised mean difference because our aim was to answer a clinical question about perceived stigma in an individual bereaved by suicide compared directly to an individual bereaved by sudden natural causes, and to an individual bereaved by sudden unnatural causes. In this clinical context, direct comparisons would be more easily interpretable than comparisons of an average effect.

We fitted a generalized ordinal regression model to ordinal outcomes (responsibility and guilt) using Stata *gologit2* commands with robust standard error [28]. This approach relaxes the proportional odds/parallel line constraints thus allowing the effect of certain variables to vary across equations.

All multivariable models included eight pre-specified confounding variables, described above, and accounted for any clustering effect at the HEI level. Models were fitted using complete case analysis, with a significance threshold of *p* = 0.05 for our primary outcome and *p* = 0.01 for secondary measures.

We conducted two distinct comparisons. The first treated the group bereaved by sudden natural causes as the reference category, to control for the sudden nature of the death. The second treated the group bereaved by sudden unnatural causes as its reference category, to control for both the sudden and the unnatural nature of the death.

To test a further pre-specified hypothesis, we added an interaction term to each of the above models, to assess whether the effect of suicide bereavement was modified by kinship (blood-related *versus* non-blood-related).

We conducted a series of *a priori* defined sensitivity analyses to assess the robustness of the study results to the various assumptions made about the missing data mechanism. These analyses included single imputation methods using best- and worst-case scenarios to impute missing values (best case substituted all missing values with lowest subscale scores; worst case substituted them with highest scores). We also assessed the robustness of findings when using more stringent inclusion criteria for the sampling strategy. These included: dropping HEIs that modified the stipulated recruitment method; those with participant numbers below the median cluster size; and those bereaved recently. Finally, we re-ran the interaction tests excluding partners, ex-partners, and non-blood relatives, to explore whether findings were unchanged using a strict definition of peer suicide.

All analyses were conducted using Stata version 12 (StataCorp., Texas, USA).

## 3 Results

**Fig. 1:**
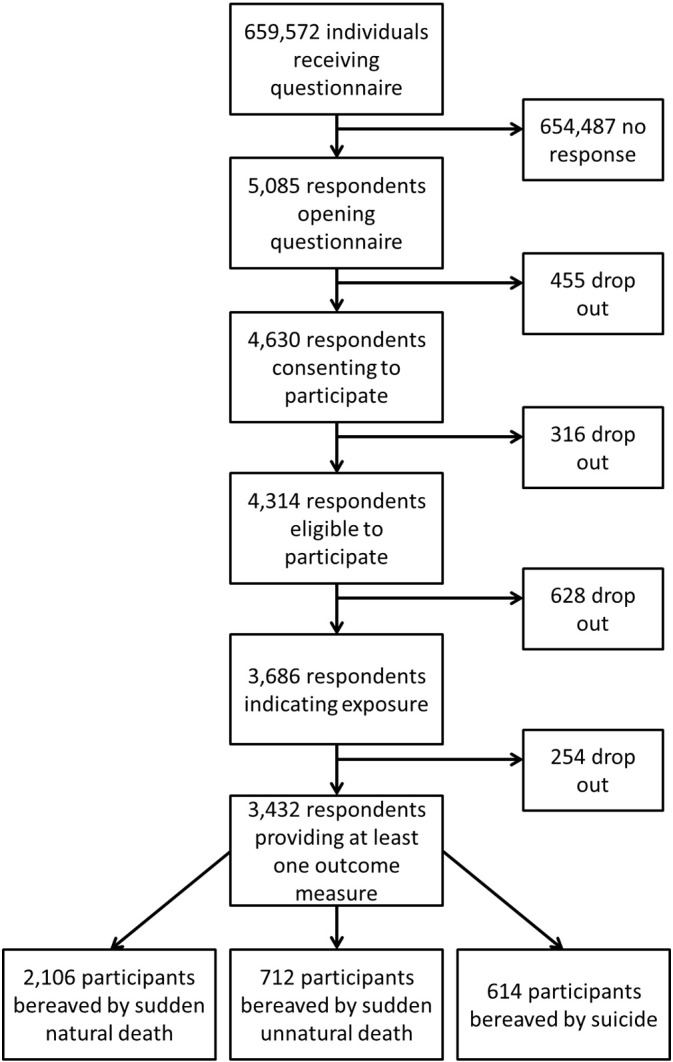
Participant flow.

A total of 5085 people of the 659,572 sampled responded to the questionnaire by clicking on the survey link, with 91% consenting to participate, and 68% (n = 3432) fulfilling eligibility (Fig. 1). The majority (61%) described the loss of a close contact due to sudden natural causes, with 21% describing loss to sudden unnatural causes, and 18% to suicide. Cluster (HEI) size varied from 3–364 participants (median = 52; inter-quartile range = 25–120). Clustering of participants within the 37 HEIs was minimal for our primary outcome, accounting for only 0.8% of the total variance (rho = 0.008), indicating low within-cluster correlation of responses. Missing data for model covariates and outcomes were less than 7%.

### 3.1 Participant characteristics

The sample was primarily female, white, and blood-related to the deceased (Table 1). Amongst non-relatives, 74% reported the death of a friend, and 11% a partner. There were no statistically significant group differences in relation to mean age, gender, self-defined ethnicity, socio-economic status, perceived level of social support, or personality disorder screen. The mean time elapsed since bereavement was 4.9 years (SD = 5.3; range = 1 day to 30 years), with no evidence of group differences, although those bereaved by sudden natural causes were significantly more likely than the other two groups to report a bereavement within the last 2 years (*p* = < 0.001) *versus* over two years previously.

### 3.2 Comparison of stigma, shame, responsibility and guilt scores

Mean stigma scores in each group ranged from 11.9 to 14.0 (Table 2). In comparison with people bereaved by sudden natural death, both the group bereaved by suicide and the group bereaved by sudden unnatural death had significantly higher stigma and shame scores (Table 2), with highest scores in those bereaved by suicide. Patterns differed for responsibility and guilt. Whilst the group bereaved by suicide had a significantly greater probability of high responsibility and guilt scores compared with those bereaved by sudden natural death, there was no evidence for differences between people bereaved by sudden unnatural death and those bereaved by sudden natural death.

Direct comparison between people bereaved by suicide and those bereaved by sudden unnatural death (Table 3), showed that the group bereaved by suicide again had significantly higher stigma and shame scores, and a significantly greater probability of high responsibility and guilt scores. These findings therefore supported our hypothesis regarding hierarchical relationships in scores, but only for stigma and shame.

Footnotes as per Table 2.

### 3.3 Blood-relatedness as a potential effect modifier

Tests for an interaction between type of bereavement and kinship to the deceased found that only the associations between suicide bereavement and guilt (using either reference category) were modified by blood-relatedness to the deceased (*p* = 0.002). Stratum-specific analyses revealed that the probability of high guilt scores in those bereaved by suicide remained significant whether the bereaved were blood-related to the deceased or not, whichever reference category was used, but it was the magnitude of the odds ratio that varied by kinship (Table 4, Table 5). These interaction tests therefore supported our hypothesis that the association between bereavement by suicide and high stigma scores applied whether the bereaved person was blood-related to the deceased or not.

### 3.4 Sensitivity analyses

The magnitude and direction of our findings were unchanged in separate sensitivity analyses imputing worst-case and best-case values for missing data, simulating potential response biases, and excluding those bereaved within 6 months. The results of our interaction tests were also unchanged when excluding the 253 respondents who reported the death of a partner, ex-partner, or non-blood relative; leaving a stratum approximating to people bereaved by peer death.

## 4 Discussion

### 4.1 Main findings

In the largest study measuring stigma after sudden loss, people bereaved by suicide had the highest levels of perceived stigma, shame, responsibility and guilt, whether compared with people bereaved by sudden unnatural death or those bereaved by sudden natural death. We also found that stigma and shame scores for those bereaved by sudden unnatural causes were higher than those for people bereaved by sudden natural causes, which supported our hypothesis regarding a stepped relationship in scores between the three groups. Our findings also clarify that friends as well as relatives describe high levels of stigma, implying that the stigma of suicide bereavement extends beyond public beliefs about tainted bloodlines. Elevated stigma scores were observed even without group differences in perceived social support, suggesting the need for a qualitative understanding of the nature of stigma perceptions.

The patterns observed are of shame co-occurring with stigma after unnatural losses, and suicide losses being characterised by all four grief components concurrently elevated. The relationship of shame to stigma suggests that shame might reflect internalisation of perceived stigma as self-stigma [30]. Indeed shame may be the key influence on helpseeking behaviour, reducing a sense of being worthy of any help, and dimming awareness of the formal and informal help on offer. In this study guilt and responsibility were investigated not so much as pathological outcomes but as a means of understanding patterns of risk differences in stigma and shame scores. The interplay between these different components of grief is likely to be complex. Whilst shame might be regarded as an affective state arising from a person's negative self-evaluation, influenced and perpetuated by perceived stigma, guilt and responsibility can be regarded as arising from a person's negative evaluation of their past behaviour, usually in relation to another's welfare [31]. Guilt and a sense of responsibility are common human experiences after transgression of one's own code of values. In a bereavement context, levels of guilt or responsibility are likely to be influenced by perceptions of the preventability and expectedness of the death, personality factors, carer burden, age, and kinship role, as well as of others' blaming attitudes.

### 4.2 Results in the context of other studies

This study is the first to show significantly higher stigma scores in people bereaved by suicide, whether compared with bereavement by sudden natural causes or non-suicide unnatural causes. It is also the first to show higher guilt scores after suicide bereavement. Previous studies using validated grief measures had found significantly higher stigma, responsibility, shame, and rejection, but not guilt scores in people bereaved by suicide when compared with bereavements by all other causes [23], [25], [26], [32], but solely an excess of rejection [33] and shame [23], [32] scores when compared specifically with those bereaved by unnatural causes of death. Our study is likely to have had greater statistical power to detect score differences between exposure groups, given its comparative sample size. Only one previous study had been conducted in Britain [25], in an older sample of mixed kinships. Age differences between the two UK samples may have influenced tendencies to express guilt. Our sampling method had wider reach than the studies sampling psychology students in single HEIs in Canada [26] or the US [32], but may have been less epidemiologically representative than those sampling parents via Canadian coroners [23], spouses via US obituaries [33], or carers via UK coroners and hospitals [25]. Our sampling of a population in which 35–37% screened positive for personality disorder may explain our unique finding of higher guilt scores in people bereaved by suicide; perhaps reflecting greater conscientiousness.

### 4.3 Strengths

This UK sample represents the largest study of stigma, shame, responsibility, or guilt scores after sudden bereavement, using distinct control groups to specify the impact of specific mortality causes. Unlike many previous such studies [3], it used a precise sampling frame, validated outcome measures [21], and distinct group comparisons, had sufficient statistical power, adjusted for any stigma deriving from pre-bereavement psychopathology, and took into account clustering of responses. Our sensitivity analyses suggest that any biases introduced by missing data or non-response had not resulted in under- or over-estimates of associations.

### 4.4 Limitations

Our sampling of young adults from HEIs created a potential for selection bias favouring higher socio-economic classes, and those with higher rates of pre-bereavement suicidal ideation. Respondents from prestigious Russell Group HEIs were over-represented (22% *versus* the expected 15%), also limiting generalisability. Contrary to expectations, our internet-mediated sampling strategy did not redress the male non-response bias typically seen in other studies of suicide bereavement [15] and psychosocial health [18]. There was also potential for response bias from the most distressed. Consequently, the results of this study may be more generalisable to young bereaved women than men, and to young adults connected with UK HEIs than those in other settings. We were unable to present a response rate due to the lack of methods for accurate estimation of the prevalence of sudden bereavement in our sampling frame, and assume that the majority of non-responders were non-bereaved. Our systematic review [3] had highlighted contradictory findings from studies investigating whether there is a differential loss of support after suicide bereavement [32], [33]. It also described the methodological problems of measuring whether there is a mismatch between the community support perceived by the suicide bereaved and the support that is objectively offered. However, our study was not able to measure levels of social support immediately before and after the loss, or enacted stigma, due to problems of recall bias and the lack of validated measures. Whilst this study measures the degree of perceived stigma after bereavement, it does not capture the nature of this stigma, although our analysis of qualitative data from this survey will address this.

### 4.5 Clinical and policy implications

Suicide prevention strategies emphasise that all health professionals should be alert to the needs of people bereaved by suicide [1], [2]. Our earlier study helped delineate what these specific needs were, finding an increased probability of suicide attempt, poor social functioning, and occupational drop-out in people bereaved by suicide [4]. The current study confirms high levels of perceived stigma, shame, responsibility and guilt in people bereaved by suicide; representing needs for interventions to reduce distress and also barriers to care. Surveys of people bereaved by suicide indicate that many feel too ashamed and guilty to seek professional help, requesting that professionals initiate early outreach [34]. Policy-makers need to design national infrastructures for outreach after suicide, giving clinicians opportunities to screen for recognised sequelae [3], [4], signpost to available resources [35], and monitor those at highest risk. By demonstrating an accepting and non-judgemental attitude, clinicians may counter the distressing experiences of perceived stigma, shame, responsibility and guilt [30]. At a structural and personal level this reinforces the message that this group are worthy of support, promoting help-seeking behaviour.

No evidence-based interventions exist to address perceived stigma after suicide bereavement, but developments are likely to be influenced by similar interventions for people stigmatised by mental illness [30], [36]. These will need to be adapted using qualitative research describing the nature and specific cultural basis of the stigma perceived by people bereaved by suicide. Rigorous trial designs will determine whether such individual or group-based interventions are effective in reducing perceived stigma, distress, and hopelessness, as well as suicidality and social and occupational difficulties.

## 5 Conclusions

This study provides clear evidence that people bereaved by suicide report the highest levels of perceived stigma, shame, responsibility and guilt compared with people bereaved by sudden natural or unnatural mortality causes. These patterns apply whether the bereaved person was a blood relative of the deceased or not, suggesting that stigmatising attitudes permeate social networks. Future research should address the lack of interventions to address perceived stigma and shame in bereaved relatives and friends after a suicide, including systems of proactive outreach to overcome barriers to help-seeking.

## Conflicts of interest

Apart from funding from the Medical Research Council, the authors have no competing interests to report.

## Figures and Tables

**Table 1:** Characteristics of participants by type of bereavement.

Participants bereaved by:	Sudden natural death (n = 2106)	Sudden unnatural death (n = 712)	Suicide (n = 614)	Total (n = 3432)	*p*-Value^a^
Gender †					
Female n (%)	1709 (81)	576 (81)	499 (81)	2784 (81)	0.982
Missing n (%)	1 (< 1)	0 (0)	0 (0)	1 (< 1)	
Age of participant (years) † mean (SD)	24.9 (6.3)	25.2 (6.3)	25.2 (6.0)	25.0 (6.3)	0.069
Self-defined ethnicity					
White n (%)	1877 (89)	645 (91)	562 (92)	3084 (90)	0.154
Non-white n (%)	228 (10)	65 (9)	52 (9)	345 (10)	
Missing n (%)	1 (< 1)	2 (< 1)	0 (0)	3 (< 1)	
Socio-economic status^b^†					
Social classes 1.1 & 1.2 n (%)	603 (29)	224 (32)	176 (29)	1003 (29)	0.179
Social class 2 n (%)	684 (33)	234 (33)	204 (33)	1122 (33)	
Social class 3 n (%)	259 (12)	77 (11)	68 (11)	404 (12)	
Social class 4 n (%)	90 (4)	34 (5)	32 (5)	156 (5)	
Social classes 5, 6, 7 & 9 n (%)	409 (19)	115 (16)	113 (18)	638 (19)	
Missing n (%)	61 (3)	27 (4)	21 (3)	109 (3)	
Educational status					
No academic qualifications n (%)	2 (< 1)	2 (< 1)	0 (0)	4 (< 1)	0.013
Attained maximum GCSE equivalent n (%)	33 (2)	8 (1)	12 (2)	53 (2)	
Attained maximum A level equivalent n (%)	929 (44)	276 (39)	243 (40)	1448 (42)	
Attained maximum degree equivalent n (%)	763 (36)	266 (37)	217 (35)	1246 (36)	
Attained post-graduate degree n (%)	373 (18)	158 (22)	142 (23)	673 (20)	
Missing n (%)	6 (< 1)	2 (< 1)	0 (0)	8 (< 1)	
Student status					
Student n (%)	1797 (85)	613 (86)	526 (86)	2936 (86)	0.822
Staff n (%)	253 (12)	78 (11)	68 (11)	399 (12)	
Both n (%)	55 (3)	21 (3)	20 (3)	96 (3)	
Missing n (%)	1 (< 1)	0 (0)	0 (0)	1 (< 1)	
Measure of current social support^a^					
No lack of perceived social support n (%)	1234 (59)	411 (58)	345 (56)	1990 (58)	0.740
Moderate lack of perceived social support n (%)	549 (26)	197 (28)	168 (27)	914 (27)	
Severe lack of perceived social support n (%)	323 (15)	102 (14)	100 (16)	525 (15)	
Missing n (%)	0 (0)	2 (< 1)	1 (< 1)	3 (< 1)	
Family history of psychiatric problems					
Yes n (%)	1243 (59)	434 (61)	412 (67)	2089 (61)	0.002
Missing n (%)	153 (7)	41 (6)	39 (6)	233 (7)	
Other family history of suicide†					
Yes n (%)	123 (6)	41 (6)	53 (7)	217 (6)	0.038
Missing n (%)	158 (8)	43 (6)	40 (7)	241 (7)	
Pre-bereavement suicidal thoughts^d^					
Yes n (%)	584 (28)	178 (25)	185 (30)	947 (28)	0.076
Missing n (%)	148 (7)	39 (6)	40 (7)	227 (7)	
Pre-bereavement suicide attempt^b^†					
Yes n (%)	125 (6)	28 (4)	49 (8)	202 (6)	0.007
Missing n (%)	154 (7)	40 (6)	40 (7)	234 (7)	
Pre-bereavement non-suicidal self-harm†					
Yes n (%)	400 (19)	121 (17)	141 (23)	662 (19)	0.016
Missing n (%)	154 (7)	40 (6)	40 (7)	234 (7)	
Post-bereavement suicidal thoughts					
Yes n (%)	911 (43)	322 (45)	299 (49)	1532 (45)	0.064
Missing n (%)	148 (7)	39 (6)	40 (7)	227 (7)	
Post-bereavement suicide attempt					
Yes n (%)	112 (5)	42 (6)	56 (9)	210 (6)	0.003
Missing n (%)	154 (7)	40 (6)	40 (7)	234 (7)	
Post-bereavement non-suicidal self-harm					
Yes n (%)	438 (20)	149 (21)	151 (25)	738 (22)	0.127
Missing n (%)	154 (7)	40 (6)	40 (7)	234 (7)	
Pre-bereavement depression†					
Yes n (%)	370 (18)	129 (18)	143 (23)	642 (19)	0.005
Missing n (%)	85 (4)	21 (3)	24 (4)	130 (4)	
Personality disorder screen positive^c^					
Yes n (%)	743 (35)	227 (32)	225 (37)	1195 (35)	0.082
Missing n (%)	131 (6)	31 (4)	33 (5)	195 (6)	
Characteristics of the bereavement					
Kinship to the deceased†					
Blood relative n (%)	1786 (85)	351 (49)	296 (48)	2433 (71)	< 0.001
Unrelated n (%)	313 (15)	356 (50)	317 (52)	980 (29)	
Missing n (%)	7 (< 1)	5 (1)	1 (< 1)	13 (< 1)	
Age of the deceased mean (SD)	55.1 (21.5)	31.0 (17.4)	31.9 (15.2)	45.9 (22.8)	< 0.001
Years since bereavement† mean (SD)	4.8 (5.3)	5.3 (5.4)	5.1 (5.0)	5.0 (5.3)	0.140

† = pre-specified confounding variable used in adjusted model.

a 2-Sided significance threshold of *p* = 0.05.

b Socio-economic status using the 5 categories from UK Office for National Statistics.

c Measure of current social support from Adult Psychiatric Morbidity Survey [19].

d 2007 UK population estimates of lifetime suicidal ideation in 16-44 year olds were 14.6-19.3%, and for suicide attempt were 4.7%-6.3% [19]. ^e^ SAPAS-SR screen for personality disorder [29].

**Table 2:** Results of regression analyses comparing Grief Experience Questionnaire subscale scores by bereavement exposure (sudden natural deaths as reference category).

Bereavement exposure group	Sudden natural death (n = 2106)		Sudden unnatural death (n = 712)					Suicide (n = 614)				
Outcome measures	Mean score (SD)	Coefficient (reference)	Mean score (SD)	Unadjusted co-efficient^a^ (95% CI)	*p*-Value⁎	Adjusted^b^ coefficient^a^ (95% CI)	*p* value⁎	Mean score (SD)	Unadjusted coefficient^a^ (95% CI)	*p-*Value⁎	Adjusted^b^ coefficient^a^ (95% CI)	*p-*Value⁎
Stigma	11.9 (3.8)	Reference	12.3 (4.0)	0.53 (0.18–0.89)	0.003	0.83 (0.47–1.19)	< 0.001	14.0 (4.3)	2.26 (1.89–2.64)	< 0.001	2.52 (2.13–2.90)	< 0.001
Shame	12.3 (3.5)	Reference	13.3 (3.6)	1.03 (0.71–1.36)	< 0.001	1.29 (0.95–1.63)	< 0.001	14.8 (4.0)	2.64 (2.30–2.98)	< 0.001	2.91 (2.56–3.27)	< 0.001

	Proportion n (%)	Odds ratio (reference)	Proportion n (%)	Unadjusted odds ratio^c^ (95% CI)	*p*-Value⁎	Adjusted^b^ odds ratio^c^ (95% CI)	*p*-Value⁎	Proportion n (%)	Unadjusted odds ratio^c^ (95% CI)	*p-*Value⁎	Adjusted^b^ odds ratio^c^ (95% CI)	*p*-Value⁎
Responsibility (highest tertile)	542 (26)	Reference	200 (28)	1.02 (0.89–1.17)	0.755	1.07 (0.92–1.24)	0.377	292 (48)	2.50 (2.08–3.01)	< 0.001	2.55 (2.06–3.16)	< 0.001
Guilt (highest tertile)	607(29)	Reference	206 (29)	0.92 (0.80–1.05)	0.214	1.01 (0.87–1.18)	0.906	261 (43)	1.86 (1.57–2.21)	< 0.001	1.98 (1.62–2.41)	< 0.001

⁎ 2-Sided significance threshold of *p* = 0.05 for primary outcome, and *p* = 0.01 for secondary measures.

a Estimate obtained using *xtmixed* command in Stata.

b Adjusted for age, gender, socio-economic status, pre-bereavement depression, pre-bereavement suicide attempt, pre-bereavement non-suicidal self-harm, other family history of suicide (excluding index bereavement), years since bereavement, and kinship to the deceased. For each model, exposure group sizes exceeded the 466 respondents required for adequate power, even when using complete case analysis.

c Estimate obtained using *gologit2* command in Stata.

**Table 3:** Results of regression analyses comparing Grief Experience Questionnaire subscale scores by bereavement exposure (sudden unnatural deaths as reference category).

Bereavement exposure group	Sudden unnatural death (n = 712)	Suicide (n = 614)			
Outcome measures	Coefficient (reference)	Unadjusted coefficient^a^ (95% CI)	*p*-Value^⁎^	Adjusted^b^ coefficient^a^ (95% CI)	*p*-Value^⁎^
Stigma	0	1.73 (1.28–2.18)	< 0.001	1.69 (1.25–2.13)	< 0.001
Shame	0	1.61 (1.19–2.02)	< 0.001	1.62 (1.22–2.03)	< 0.001

	Odds ratio (reference)	Unadjusted odds ratio^c^ (95% CI)	*p*-Value^⁎^	Adjusted^b^ odds ratio^c^ (95% CI)	*p*-Value^⁎^
Responsibility (highest tertile)	1	2.45 (1.90–3.17)	< 0.001	2.39 (1.83–3.12)	< 0.001
Guilt (highest tertile)	1	2.03 (1.58–2.60)	< 0.001	1.96 (1.52–2.52)	< 0.001

**Table 4:** Table showing results of stratification on kinship for guilt (sudden natural deaths as reference category).

Bereavement exposure	Sudden natural death	Sudden unnatural death	Suicide
Outcome measures	(n = 2106)	(n = 712)	(n = 614)
AOR for highest guilt tertile (overall)	Reference	1.01 (0.87–1.18) (*p* = 0.906)	1.98 (1.62–2.41) (*p* = < 0.001)

Stratified by kinship			
AOR for highest guilt tertile in non-relatives	Reference	1.50 (1.20–1.87) (*p* = < 0.001)	2.76^a^ (2.09–3.64) (*p* = < 0.001)
AOR for highest guilt tertile in relatives	Reference	0.84 (0.63–1.11) (*p* = 0.222)	1.74 (1.31–2.31) (*p* = < 0.001)

AOR = adjusted odds ratio (95% CI).

a In a sensitivity analysis confined to peers only AOR = 2.92; 95% CI = 2.07–4.14; p = < 0.001.

**Table 5:** Table showing results of stratification on kinship for guilt (sudden unnatural deaths as reference category).

Bereavement exposure	Sudden unnatural death	Sudden natural death	Suicide
Outcome measures	(n = 712)	(n = 2106)	(n = 614)
AOR for highest guilt tertile (overall)	Reference	0.99 (0.85–1.16) (*p* = 0.906)	1.96 (1.52–2.52) (*p* = < 0.001)

Stratified by kinship			
AOR for highest guilt tertile in non-relatives	Reference	0.67 (0.54–0.83) (*p* = < 0.001)	1.84^a^ (1.40–2.43) (*p* = < 0.001)
AOR for highest guilt tertile in relatives	Reference	1.19 (0.90–1.59) (*p* = 0.222)	2.08 (1.32–3.28) (*p* = 0.002)

AOR = adjusted odds ratio (95% CI).

a In a sensitivity analysis confined to peers only AOR = 2.04; 95%CI = 1.49–2.80; *p* = < 0.001).

## Box 1 GEQ stigmatization subscale items. Stem: Since the death how often did you…

1. feel like a social outcast?
2. feel like no-one cared to listen to you?
3. feel that neighbours and friends did not offer enough concern?
4. feel avoided by friends?
5. think people were gossiping about you or the person?
6. think that others didn't want you to talk about the death?
7. feel somehow stigmatised by the death?
8. feel like people were probably wondering about what kind of personal problems you and the person had experienced?
9. think that people were uncomfortable offering their condolences to you?
10. feel like the death somehow reflected negatively on you or your family?

Alt-text: Box 1

## Appendix A Supplementary data

Supplementary material 1 Survey instrument: UCL Bereavement Study questionnaire. Supplementary material 1

Supplementary material 2 STROBE statement. Supplementary material 2
