# Supplementary material for: The stigma perceived by people bereaved by suicide and other sudden deaths: A cross-sectional UK study of 3432 bereaved adults
Source: J Psychosom Res. 2016 Aug;87:22–9. doi: 10.1016/j.jpsychores.2016.05.009 (PMC4988532; doi:10.1016/j.jpsychores.2016.05.009)
Supplement: Supplementary material 1 — Survey instrument: UCL Bereavement Study questionnaire. [file mmc1.docx]

**
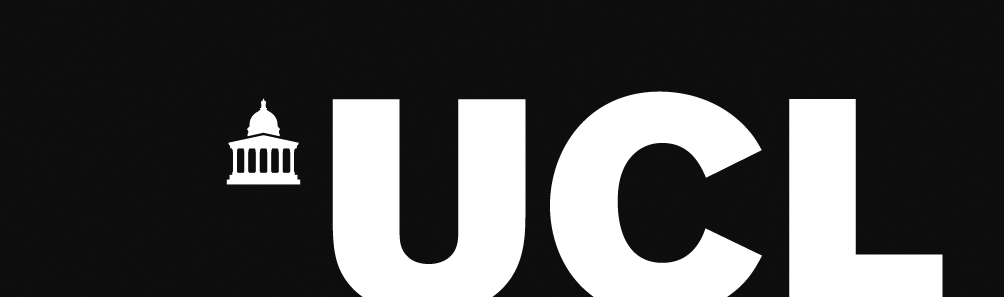
** [
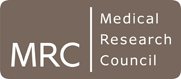
](http://www.mrc.ac.uk/)

**UCL Bereavement Study questionnaire**

### Part 1

Thank you for taking part.

*To continue to the survey click****Next****at the bottom of this page.*

**Study aims**

This survey will help us to understand the impact of sudden bereavement. The results will be used to improve services for bereaved people.

**How to complete the survey**

In Part 1 the responses are by ticking boxes and should take about 5-10 minutes. In Part 2 the responses are given in free text boxes for you to provide as much detail as you wish, and usually takes between 5 and 15 minutes.

**How to save progress and take a break**

Clicking the Save button gives you instructions on how to return later. The program asks for your email address to send you a 'return ticket' link. Your email address will not be added to the data and this protects your confidentiality.

**Anonymity**

The survey is anonymous. We can't link answers to you unless you give your name. If you do give your name or email we will not pass this to any organisation outside our research team. All data will be collected and stored in accordance with the Data Protection Act 1998.

**In case of difficulty**

Completing this questionnaire may not be easy for some people and some questions may evoke difficult memories. For a list of sources of support please bookmark the study website: [www.ucl.ac.uk/psychiatry/bereavementstudy](http://www.ucl.ac.uk/psychiatry/bereavementstudy)

**Further information**

If you have questions about the questionnaire click on [this link](mailto:bereavementstudy@ucl.ac.uk?subject=Bereavement%20Study%20enquiry) (to email address: bereavementstudy@ucl.ac.uk) to contact us.

#### Consent to participate:

Question 1

Before you start this study we need to check that we have your informed consent to participate. Once you have read the UCL Bereavement Study information sheet and consent form (at this hyperlink) please tick the box below if you agree with the following statement. 

I understand the aim of this study and agree that its anonymised results are to be used for scientific purposes and further analyses.

□ Yes – I agree

Question 2

This survey has been sent to you by (*name of higher education institution from list of 37 participating)*. 

Which one of the following describes your connection to (insert name of HEI)?

□ a student at (*HEI name*)

□ a member of staff at (*HEI name*)

□ a member of staff at (*HEI name*) who is also registered there as a student

□ None of the above (branch to end of questionnaire thanking respondent for their interest)

#### Demographic information

These first few questions in Part 1 are to find out some of your characteristics. It will help us compare your answers with those of other people who are similar to you.

Question 3

What is your gender?

□ Male

□ Female

Question 4

What is your age?

(drop-down ages 18-40)

□ My age is outside this age range.  (branch to end of questionnaire thanking respondent for their interest and reminding them of inclusion criteria)

Please note that the age range for this study is 18 to 40. This in no way implies that bereavement has a lesser impact in other age-groups, but allows us to focus on a specific sub-group which has tended to be under-represented in work of this kind. If your age is outside this range we cannot use your responses in the data analysis, but thank you for having volunteered your time. 

For a list of support services, and for further information on the progress of this study, see the [UCL Bereavement Study website](http://www.ucl.ac.uk/psychiatry/bereavementstudy): link to [www.ucl.ac.uk/psychiatry/bereavementstudy](http://www.ucl.ac.uk/psychiatry/bereavementstudy)

Question 5

What is your work status? Please tick one of the options below.

□ full-time paid work (> 30 hours per week)

□ part-time paid work (< 30 hours per week)

□ full-time student

□ full-time student with part-time job

□ part-time student

□ part-time student with part-time job

□ Other - please state

Question 6

Over the last 12 months how many days have you been absent from work, study, training or care responsibilities? (space for entering numerals 0-365 days)

Question 7 – for staff

Please state your occupation, including any managerial responsibilities you may have.

(Free text: coded into ONS categories)

Question 8 – for students

Please state the occupation of your highest-earning parent, or the person who supports you financially. If he or she is retired or unemployed, give their most recent occupation.

(Free text: coded into ONS categories)

Question 9

What is your highest level of education? Please tick the option which represents your highest level of exam achievement.

□ no academic qualifications

□ lower secondary education (eg. GCSEs, O levels, CSEs)

□ higher secondary education (eg. A levels, Highers, IB, Access course)

□ university degree (undergraduate)

□ higher university degree (post-graduate)

□ other - please state

Question 10

What is your ethnicity?  Please tick one of the options below

□ White British

□ White Irish

□ Other White groups

□ Asian or Asian British: Indian

□ Asian or Asian British: Pakistani

□ Asian or Asian British: Bangladeshi

□ Asian or Asian British: all other

□ Black or Black British: Caribbean

□ Black or Black British: African

□ Black or Black British: all other

□ Mixed race: White and Black Caribbean

□ Mixed race: White and Black African

□ Mixed race: White and Asian

□ Mixed race: all other

□ Chinese

□ Other ethnic groups

□ Unable to respond

Question 11

What is your religion? Please tick one of the options below.

□ No religious affiliation but holding spiritual beliefs

□ Atheist

□ Agnostic

□ Buddhist

□ Hindu

□ Jewish

□ Muslim

□ Sikh

□ Christian - Catholic

□ Christian - Protestant

□ Christian - other Christian group

□ Other - please specify in this box

Question 12

What is your marital status? Please tick one of the options below.

□ single

□ co-habiting

□ married/civil union

□ divorced

□ separated

□ widowed

Question 13

How many children do you have?

(space for entering numerals)

Question 14

What is your current living situation? Please tick one of the options below.

□ alone

□ living with spouse/partner

□ single parent living with children

□ living with parents

□ living with other relatives

□ sharing accommodation with non-relatives

□ student hall of residence or student hostel

□ temporary hostel or B&B accommodation

□ homeless

□ other - please specify

#### Social support

The next few questions are about people you feel close to, including relatives, friends and acquaintances.

Question 15

First of all we would like to ask you about the people that you live with. 
How many adults who live with you do you feel close to?

(space for entering numerals 0-99)

Question 16

Now we would like to ask about people you feel close to who do not live with you. 
How many relatives aged 16 or over, who do not live with you, do you feel close to?

(space for entering numerals 0-99)

Question 17

How many friends or acquaintances who do not live with you would you describe as close or good friends?

(space for entering numerals 0-99)

Question 18

Thinking about all of the people who do not live with you, and whom you feel close to or regard as good friends, how many did you communicate with in the last week?

(space for entering numerals 0-99)

We would now like you to think about your family and friends. (By family we mean those who live with you as well as those elsewhere). Here are some comments people have made about their family and their friends. For each statement, please say whether it is not true, partly true or certainly true for you.

Question 19

There are people I know amongst my family and friends who do things to make me happy.

□ Not true

□ Partly true

□ Certainly true

Question 20

There are people I know amongst my family and friends who make me feel loved.

□ Not true

□ Partly true

□ Certainly true

Question 21

There are people I know amongst my family and friends who can be relied on, no matter what happens.

□ Not true

□ Partly true

□ Certainly true

Question 22

There are people I know amongst my family and friends who would see that I am taken care of if I needed to be.

□ Not true

□ Partly true

□ Certainly true

Question 23

There are people I know amongst my family and friends who accept me just as I am.

□ Not true

□ Partly true

□ Certainly true

Question 24

There are people I know amongst my family and friends who make me feel an important part of their lives.

□ Not true

□ Partly true

□ Certainly true

Question 25

There are people I know amongst my family and friends who give me support and encouragement.

□ Not true

□ Partly true

□ Certainly true

#### Past bereavements

Question 26

Since you were aged 10 have you experienced a sudden bereavement of someone close to you due to any of the following? 

Please tick *all* those that apply to you.

□ sudden natural death (eg. cardiac arrest, epileptic seizure, stroke)  (branch to questions 28 and 29)

□ sudden un-natural death (eg. road crash, murder or manslaughter, work accident) (branch to questions 28 and 29)

□ suicide (branch to question 27)

Question 27

We would like to hear more about the impact of your bereavement by suicide. 

The rest of the questions in this survey relate to the impact of a suicide on your everyday functioning and other aspects of your life. 

If you have been bereaved by suicide more than once please answer the rest of this questionnaire in relation to one person - the person to whom you felt closest. 

What gender was this person?

□ Male

□ Female

Question 28

The rest of the questions in this survey relate to the impact of one specific sudden bereavement on your everyday functioning and other aspects of your life. 

If you have been bereaved suddenly more than once please answer the rest of this questionnaire in relation to one person - the person to whom you felt closest. 

How did this person die?

□ sudden natural death (eg. cardiac arrest, epileptic seizure, stroke)

□ sudden un-natural death (eg. road crash, murder or manslaughter, work accident)

□ suicide

Question 29

What gender was this person?

□ Male

□ Female

Question 30

Please give an estimate of how old you were when this person died? 

Remember from the website that we are including sudden unexpected bereavements you may have experienced after you reached the age of 10. The survey starts at the age of 10 because children tend to react to bereavement in different ways to adolescents or adults, and because there may be difficulties remembering events in childhood.

(drop-down ages 10-40).

□ I was under 10 at the time of that person’s death (branch to end of questionnaire thanking respondent for their interest and reminding them of inclusion criteria)

*If the bereavement was before this age we will be unable to include your responses in our analysis but thank you for having volunteered your time. For further information on the progress of this study see:* [www.ucl.ac.uk/psychiatry/bereavementstudy](http://www.ucl.ac.uk/psychiatry/bereavementstudy)

Question 31

About how long ago did this person die? (space) years ago

Question 32

Approximately how old was this person at the time of their death?

(space) years old

In the case of infant death please use this space to give their age: (Free text)

#### Your relationship to the person who died

Question 33

What relation was this person to you?  Please tick one of the following options. 
i.e. He/she was my...

□ brother

□ sister

□ father

□ mother

□ son

□ daughter

□ partner or spouse

□ ex-partner or ex-spouse

□ grandparent

□ close friend

□ close colleague or client

□ cousin

□ niece or nephew

□ uncle or aunt

□ uncle by marriage or aunt by marriage

□ brother-in-law or sister-in-law

□ mother-in-law or father-in-law

□ other – please state (Free text)

Please specify here if they were a half-, step-, or adoptive relative or a relative by marriage: (Free text)

Question 34

In the year before their death on average how often were you in contact with them? 
This would include face-to-face meetings as well as telephone calls, text messages, emails, cards, letters, and contact via social networking sites.

□ daily

□ weekly

□ monthly

□ every 2 to 3 months

□ every 6 months

□ yearly

□ not at all during that year

Question 35

Approximately how long before their death had you known this person for?

For (space) years

Question 36

At the time of their death how emotionally close did you feel to this person?

Not close at all 1 □ 2 □ 3 □ 4 □ 5 □ As close as any relationship I've had before or since

Question 37

If your relationship with them had previously been closer or more distant, please try and rate how close the relationship had been previously using the same scale. 
If the relationship was no different previously you can just click *N/A*.

Not close at all 1 □ 2 □ 3 □ 4 □ 5 □ As close as any relationship I've had before or since □ N/A

#### The emotional impact of a bereavement

The following questions are to find out about the types of feelings you may have had since the death. 

You may find that some of the questions asked do not apply to you. For these you should tick 'Never'. 

For those questions that you do identify with please try to judge, as best you can, how frequently you have experienced this feeling since the death.

Question 38

How often did you think that people were uncomfortable offering their condolences to you?

□ Never

□ Rarely

□ Sometimes

□ Often

□ Almost always

Question 39

How often did you avoid talking about the negative or unpleasant parts of your relationship?

□ Never

□ Rarely

□ Sometimes

□ Often

□ Almost always

Question 40

How often did you feel avoided by friends?

□ Never

□ Rarely

□ Sometimes

□ Often

□ Almost always

Question 41

How often did you think that others didn't want you to talk about the death?

□ Never

□ Rarely

□ Sometimes

□ Often

□ Almost always

Question 42

How often did you feel like no-one cared to listen to you?

□ Never

□ Rarely

□ Sometimes

□ Often

□ Almost always

Question 43

How often did you feel that friends, neighbours and family did not offer enough concern?

□ Never

□ Rarely

□ Sometimes

□ Often

□ Almost always

Question 44

How often did you feel like a social outcast?

□ Never

□ Rarely

□ Sometimes

□ Often

□ Almost always

Question 45

How often did you think people were gossiping about you or that person?

□ Never

□ Rarely

□ Sometimes

□ Often

□ Almost always

Question 46

How often did you feel like people were probably wondering about what kind of personal problems you and that person had experienced?

□ Never

□ Rarely

□ Sometimes

□ Often

□ Almost always

Question 47

How often did you feel like others may have blamed you for the death?

□ Never

□ Rarely

□ Sometimes

□ Often

□ Almost always

Question 48

How often did you feel like the death somehow reflected negatively on you or your family?

□ Never

□ Rarely

□ Sometimes

□ Often

□ Almost always

Question 49

How often did you feel somehow stigmatised by the death?

□ Never

□ Rarely

□ Sometimes

□ Often

□ Almost always

Question 50

How often did you think of times before the death when you could have made the person's life more pleasant?

□ Never

□ Rarely

□ Sometimes

□ Often

□ Almost always

Question 51

How often did you wish that you hadn't said or done certain things during your relationship with the person?

□ Never

□ Rarely

□ Sometimes

□ Often

□ Almost always

Question 52

How often did you feel like there was something very important you wanted to make up to the person?

□ Never

□ Rarely

□ Sometimes

□ Often

□ Almost always

Question 53

How often did you feel like maybe you didn't care enough about the person?

□ Never

□ Rarely

□ Sometimes

□ Often

□ Almost always

Question 54

How often did you feel somehow guilty after the death of the person?

□ Never

□ Rarely

□ Sometimes

□ Often

□ Almost always

Question 55

How often did you feel like the person had some kind of complaint against you at the time of the death?

□ Never

□ Rarely

□ Sometimes

□ Often

□ Almost always

Question 56

How often did you feel that, had you somehow been a different person, the person would not have died?

□ Never

□ Rarely

□ Sometimes

□ Often

□ Almost always

Question 57

How often did you feel that you had made the person unhappy long before the death?

□ Never

□ Rarely

□ Sometimes

□ Often

□ Almost always

Question 58

How often did you feel as though problems you and that person had together contributed to an untimely death?

□ Never

□ Rarely

□ Sometimes

□ Often

□ Almost always

Question 59

How often did you avoid talking about the death of the person?

□ Never

□ Rarely

□ Sometimes

□ Often

□ Almost always

 Question 60

How often did you feel uncomfortable revealing the cause of the death?

□ Never

□ Rarely

□ Sometimes

□ Often

□ Almost always

Question 61

How often did you feel embarrassed about the death?

□ Never

□ Rarely

□ Sometimes

□ Often

□ Almost always

Question 62

How often did you not mention the death to people you met casually?

□ Never

□ Rarely

□ Sometimes

□ Often

□ Almost always

Question 63

How often did you tell someone that the cause of death was something different than what it really was?

□ Never

□ Rarely

□ Sometimes

□ Often

□ Almost always

#### Accessing help

We are now interested in finding out about the help you received or were offered after the death. 
If you wish to give more detail there are further questions about this in **Part 2.**

Question 64

How long after the death did you receive help that was valuable to you?

□ Within a day

□ Within a week

□ Within a month

□ Within 6 months

□ Within a year

□ Over a year

□ At no time

Question 65

What help did you receive after the death? Please tick all those that apply:

□ None

□ Police

□ Funeral director

□ Coroner’s service

□ NHS services (doctor, nurse, therapist, counsellor)

□ Private counsellor or therapist

□ Voluntary sector services (helpline, counsellor)

□ Help from friends, family and neighbours

□ Self-help from a website, book or leaflet

□ Other – please state: (Free text)

We are now interested in finding out about your emotional health.

Question 66

Have you ever, except in the last 6 months, had nearly two weeks or longer when nearly every day you felt sad, empty or depressed for most of the day?

□ Yes (branch to question 67)

□ No

Question 67

If Yes, at about what age did these feelings of being sad, empty or depressed first occur? (space) years old

Question 68

Have you ever, except in the last 6 months, had 2 weeks or longer when you lost interest in most things like work, hobbies and other things that you usually enjoyed?

□ Yes (branch to question 69)

□ No

Question 69

If Yes, at what age did these feelings of having lost interest in most things first occur?

(space) years old

Question 70

During the last 30 days, about how often did you feel tired out for no good reason?

□ None of the time

□ A little of the time

□ Some of the time

□ Most of the time

□ All of the time

Question 71

During the last 30 days, about how often did you feel nervous?

□ None of the time

□ A little of the time

□ Some of the time

□ Most of the time

□ All of the time

Question 72

During the last 30 days, about how often did you feel so nervous that nothing could calm you?

□ None of the time

□ A little of the time

□ Some of the time

□ Most of the time

□ All of the time

Question 73

During the last 30 days, about how often did you feel hopeless?

□ None of the time

□ A little of the time

□ Some of the time

□ Most of the time

□ All of the time

Question 74

During the last 30 days, about how often did you feel restless or fidgety?

□ None of the time

□ A little of the time

□ Some of the time

□ Most of the time

□ All of the time

Question 75

During the last 30 days, about how often did you feel so restless you could not sit still?

□ None of the time

□ A little of the time

□ Some of the time

□ Most of the time

□ All of the time

Question 76

During the last 30 days, about how often did you feel depressed?

□ None of the time

□ A little of the time

□ Some of the time

□ Most of the time

□ All of the time

Question 77

During the last 30 days, about how often did you feel that everything was an effort?

□ None of the time

□ A little of the time

□ Some of the time

□ Most of the time

□ All of the time

Question 78

During the last 30 days, about how often did you feel so sad that nothing could cheer you up?

□ None of the time

□ A little of the time

□ Some of the time

□ Most of the time

□ All of the time

Question 79

During the last 30 days, about how often did you feel worthless?

□ None of the time

□ A little of the time

□ Some of the time

□ Most of the time

□ All of the time

Question 80

In the last month how often have you had intense feelings of emotional pain, sorrow, or pangs of grief related to the person who died?

□ Not at all

□ At least once this month

□ At least once a week

□ At least once a day

□ Several times a day

#### Personality style

The following 8 questions are about your personality - the way you typically think, feel or behave.

Question 81

In general, do you have difficulty making and keeping friends?

□ Yes

□ No

Question 82

Would you normally describe yourself as a loner?

□ Yes

□ No

Question 83

In general, do you trust other people? Please base your answer on whether you think that the description applies *most of the time* and in most situations.

□ Yes

□ No

Question 84

Do you normally lose your temper easily?

□ Yes

□ No

Question 85

Are you normally an impulsive sort of person?

□ Yes

□ No

Question 86

Are you normally a worrier?

□ Yes

□ No

Question 87

In general, do you depend on others a lot?

□ Yes

□ No

Question 88

In general, are you a perfectionist?

□ Yes

□ No

#### Your day-to-day life

The next 8 questions measure how you currently handle everyday life and relationships. 

Each one is presented as a statement. Please look at each statement and tick the reply that comes closest to how you have been **over the last fortnight**.

Question 89

I complete my tasks at work and home satisfactorily.

□ Most of the time

□ Quite often

□ Sometimes

□ Not at all

Question 90

I find my tasks at work and at home very stressful.

□ Most of the time

□ Quite often

□ Sometimes

□ Not at all

Question 91

I have no money problems.

□ No problems at all

□ Slight worries only

□ Definite problems

□ Very severe problems

Question 92

I have difficulties in getting and keeping close relationships.

□ Severe difficulties

□ Some problems

□ Occasional problems

□ No problems at all

Question 93

I have problems in my sex life.

□ Severe problems

□ Moderate problems

□ Occasional problems

□ No problems at all

Question 94

I get on well with my family and other relatives.

□ Yes, definitely

□ Yes, usually

□ No, some problems

□ No, severe problems

Question 95

I feel lonely and isolated from other people.

□ Almost all the time

□ Much of the time

□ Not usually

□ Not at all

Question 96

I enjoy my spare time.

□ Very much

□ Sometimes

□ Not often

□ Not at all

Question 97

In relation to your education, have you ever had to drop out of a course at school, college or university?

□ Yes (branch to question 98)

□ No

□ Not applicable

Question 98

If yes was this:

□ before the bereavement?

□ after the bereavement?

□ both before and after the bereavement?

Question 99

In relation to your employment history, have you ever:

- been made redundant?
- been disciplined?
- resigned from a job for negative reasons?
- been given notice from employment?

□ Yes (branch to question 100)

□ No

□ Not applicable

Question 100

If yes was this:

□ before the bereavement?

□ after the bereavement?

□ both before and after the bereavement?

The following questions are about times in your life when you might have felt low and hopeless, with negative thoughts about your future.

Question 101

Have you ever thought that life was not worth living?

□ No

□ Yes (branch to question 102)

Question 102

If you have ever thought that life was not worth living, was this:

□ before the bereavement?

□ after the bereavement?

□ both before and after the bereavement?

Question 103

Have you ever wished that you were dead?

□ No

□ Yes (branch to question 104)

Question 104

If you have ever wished that you were dead, was this

□ before the bereavement?

□ after the bereavement?

□ both before and after the bereavement?

Question 105

Have you ever thought of taking your life, even though you would not actually do it?

□ No

□ Yes (branch to question 106)

Question 106

If you have ever thought of taking your life, was this:

□ before the bereavement?

□ after the bereavement?

□ both before and after the bereavement?

Question 107

Have you ever made an attempt to take your life, by taking an overdose of tablets or in some other way?

□ No

□ Yes (branch to question 108)

Question 108

If you have ever made an attempt to take your life, by taking an overdose of tablets or in some other way, was this:

□ before the bereavement?

□ after the bereavement?

□ both before and after the bereavement?

Question 109

Have you ever deliberately harmed yourself in any way but not with the intention of killing yourself?

□ No

□ Yes (branch to question 110)

Question 110

If you have ever deliberately harmed yourself in any way, but not with the intention of killing yourself, was this:

□ before the bereavement?

□ after the bereavement?

□ both before and after the bereavement?

#### Seeking help

Question 111

If you have harmed yourself since the bereavement did you seek help from anyone?

□ Yes (branch to question 112)

□ No

□ Not applicable

Question 112

Who did you try to get help from?  Please tick all those that apply.

□ a friend

□ a member of your family

□ your GP/family doctor

□ the local hospital

□ someone else ? Please specify: (Free text).

The next few questions relate to the psychological health of other people in your family.

Question 113

Has anyone in your family suffered from an anxiety disorder, a depressive disorder (including postnatal depression), had drug or alcohol problems, or other psychological or emotional difficulties?

□ Yes (branch to question 114)

□ No

Question 114

Please use this box to indicate which members of your family have had psychological or emotional difficulties, specifying whether they were blood relatives or not. If your earlier responses about bereavement related to a family member, and this question applies to that person, please include them here too. (Free text)

Question 115

Have any of your blood relatives died by suicide? 
If your earlier responses about bereavement related to the suicide of a relative please tick **Yes** as the computer programme cannot add this information automatically.

□ Yes (branch to question 116)

□ No

Question 116

If yes please use the box below to indicate what relative they were to you (e.g. father, grandfather, aunt, etc). (Free text)

These final few questions in Part 1 are about your own psychological health.

Question 117

Have you ever had an anxiety disorder, a depressive disorder, drug or alcohol problems, or other mental health difficulties?

□ Yes (branch to question 118)

□ No

□ Don’t know

Question 118

If you have had psychological or emotional difficulties, have you ever had help for this from any of the following:

- general practitioner?
- practice nurse?
- practice counsellor?
- a psychiatrist in an out-patient appointment?

□ Yes

□ No

□ Don’t know

□ Not applicable

Please use the free text below if you wish to give further details: (Free text)

Question 119

Have you ever been an in-patient in an acute mental health ward?

□ Yes

□ No

Please use the free text box below if you wish to give further details: (Free text)

Question 120

This is the final question in Part 1.  To date, at what stage after the bereavement do you feel that you have been most affected by it?

□ immediately afterwards

□ up to a week

□ up to a month

□ up to 6 months

□ up to a year

□ up to 3 years

□ over 3 years

### Part 2

Thank you for your answers so far. Here in **Part 2** the free text boxes are for you to tell us in your own words about the areas of your life that might have been affected.

- Please give as much or as little detail as you wish to.
- If a question does not apply to you, just skip it.
- At any stage you can click **Save** in order to return and continue at another time.

#### Relationships

Question 121

In what way, if any, has your relationship with a partner, or with potential partners, changed since the bereavement? 
Remember that if this or any other question does not apply to you, just skip to the next one.

Question 122

What about relationships with close friends, or with potential close friends?

Question 123

In what way, if any, have relationships within your immediate family (parents, brothers, sisters, children) changed since the bereavement?

Question 124

What about relationships with members of the wider family (cousins, aunts, uncles, nephews, nieces, grandparents)?

Question 125

If there are other ways in which you have withdrawn from those around you or grown closer to them, please use this space to give details. (Free text)

#### Education and work

Question 126

In what way, if any, has the bereavement affected your educational progress?

Question 127

What about your work performance?

#### Other aspects of everyday life

Question 128

In what way, if any, has the bereavement affected your drinking habits or your use of unprescribed drugs? (Unprescribed drugs include illicit drugs as well as medications used above their prescribed limits.)

Question 129

In what way, if any, has the bereavement affected your finances?

Question 130

In what way, if any, has the bereavement affected your spiritual beliefs?

Question 131

What information about the circumstances of their death, if any, did you not find out about until later?

Question 132

In what situations, if any, have you avoided discussing the death, or noticed that others avoid the subject?

Question 133

In what situations, if any, have you hidden your grief to protect yourself and others?

Question 134

Is the person who died still talked about by those who knew them? 
In your answer you may want to consider:

- Whether anyone avoids talking about them.
- Whether anyone has made negative comments about them or the way they died.
- What opportunities you have had to share memories of them.

Question 135

To what extent has their death made you fear that you may die in a similar way?

#### Immediately after the death

Question 136

If you attended a funeral or memorial service for the person who died, what was your experience of this?

Question 137

If an inquest was held what was your experience of this, and your reaction to the verdict?

Question 138

Please describe any positive or negative experiences you may have had after the death in relation to the following:

- police force
- funeral directors
- coroner's office
- healthcare staff
- press reporting on the death

#### Help received

Question 139

What are your views on any help you were offered or not offered? 
In your response you may wish to comment on:

- how helpful or unhelpful any support was
- what help you wish you had been offered and at what stage
- why certain people did not offer their support

(Free text)

Question 140

After the death did it feel as though support was available to other people close to that person but not to yourself? 
For example this may have been because:

- you hid your grief
- others were not aware that you had a close relationship with this person
- the support you wanted was not available

#### Future work and feedback

You have reached the end of the questionnaire. Thank you very much for your time. 

We are also inviting some people who have completed this survey to participate in a face-to-face **interview**. This gives us a chance to hear more about your personal experiences of bereavement. 

The interview lasts up to an hour. In London these will be held at UCL (Torrington Place, London WC1), but arrangements for sites outside London are to be confirmed. Further information is available on the study website: [www.ucl.ac.uk/psychiatry/bereavementstudy](http://www.ucl.ac.uk/psychiatry/bereavementstudy) 

Question 141

If you would be willing to be contacted about volunteering for an interview please type your email address and/or telephone number in the space below. These details will **not** be passed on to anyone outside this research team. 

Please note that if you do volunteer for an interview that you are not committed to this, and can withdraw this decision at any time.

Email and/or telephone: (Free text)

Question 142 - Future work: 

We also hope to conduct a follow-up study in a few years’ time to explore whether there are any changes in how people adjust to a bereavement over time. 

If you are willing to be contacted about participating in this future study please supply contact details which will be reliable for a period of approximately 5 years. 
If your email address is likely to change you may prefer to give a postal address or telephone number. If we contact you by post the envelope will be marked *only* with your name and address, and will be labelled Private and Confidential. 
If you know your NHS number this is also a reliable way of our team being able to contact you by post. 

Volunteering for this follow-up study is entirely optional. If you do provide contact details but later decide not to participate, you are free to withdraw at any time. You do not have to give any reason for withdrawing. 

At no point will your name or contact details be passed on to anyone outside the research team.

Email/Telephone/Postal address/NHS number: (Free text)

Thank you for participating in this questionnaire.

Question 143 - Communication of the study's results: 

Once the results have been analysed they will be available on the UCL Bereavement Study website.
If you would like the results to be emailed to you please type your email address in the box below. 

Please note that: 
1) Your email address will not be visible to others when the study results are emailed out. 
2) When the responses to this questionnaire are analysed your email address will be removed so your anonymity is protected. 
3) Your name or contact details will not be passed on to anyone outside this research team.

Email: (Free text)

Clicking on the Finish button (bottom right) will end your questionnaire and bring you directly to the university's counselling service website. This is your opportunity to go back and review your responses if you wish to.
