# Supplementary material for: The stigma perceived by people bereaved by suicide and other sudden deaths: A cross-sectional UK study of 3432 bereaved adults
Source: J Psychosom Res. 2016 Aug;87:22–9. doi: 10.1016/j.jpsychores.2016.05.009 (PMC4988532; doi:10.1016/j.jpsychores.2016.05.009)
Supplement: Supplementary material 2 — STROBE statement. [file mmc2.docx]

**STROBE checklist for UCL Bereavement Study**

Checklist of items that should be included in reports of ***cross-sectional studies***:

<http://www.strobe-statement.org/index.php?id=available-checklists>

|  | Item No | Recommendation |
| --- | --- | --- |
| **Title and abstract** | 1 | **(*a*) Indicate the study’s design with a commonly used term in the title or the abstract:** abstract indicates that we conducted a national cross-sectional study |
|  |  | **(*b*) Provide in the abstract an informative and balanced summary of what was done and what was found:** abstract outlines our hypothesis, exposures and outcomes, an adjusted coefficients for the associations hypothesised |
| Introduction | | |
| Background/rationale | 2 | **Explain the scientific background and rationale for the investigation being reported:** Our introduction outlines the policy context, including key research references, and highlights the lack of evidence to support current suicide prevention strategy, and the particular need to investigate stigma. |
| Objectives | 3 | **State specific objectives, including any prespecified hypotheses:**  Objectives and primary hypothesis stated in the Abstract and Introduction. Our objective was to conduct a population-based survey comparing the impact of different modes of sudden bereavement on non-fatal suicide-related outcomes, other clinical and occupational outcomes, and stigma. Our hypothesis was that suicide bereavement among young UK-based adults, compared with bereavement by other causes of sudden death, was associated with high levels of stigma. Further pre-specified hypotheses are stated in the Introduction. |
| Methods | | |
| Study design | 4 | **Present key elements of study design early in the paper:** Cross-sectional survey stated in Methods. |
| Setting | 5 | **Describe the setting, locations, and relevant dates, including periods of recruitment, exposure, follow-up, and data collection**: Method describes emailing sample of 37 HEIs in 2010 for cross-sectional data collection. Acknowledgement section details the locations and range of HEIs. |
| Participants | 6 | **Give the eligibility criteria, and the sources and methods of selection of participants:** Eligibility criteria described as: people aged 18-40 who had experienced sudden bereavement of a close friend or relative after ten years of age. |
| Variables | 7 | **Clearly define all outcomes, exposures, predictors, potential confounders, and effect modifiers. Give diagnostic criteria, if applicable.**  All 4 outcomes described, providing citation for validation studies.  Exposure clearly defined. Eight pre-specified confounding variables defined and justified. Kinship defined as a potential effect modifier. |
| Data sources/ measurement | 8* | **For each variable of interest, give sources of data and details of methods of assessment (measurement). Describe comparability of assessment methods if there is more than one group:**  Questionnaire development and content described. Same instrument used for all exposure groups. |
| Bias | 9 | **Describe any efforts to address potential sources of bias:** We describe how we followed-up non-responding HEIs to ensure a diverse representation of HEIs, and how we masked participants to the study hypothesis. We also describe a decision to use two-tailed analysis to reduce inductive bias. |
| Study size | 10 | **Explain how the study size was arrived at:** We based our sample size calculation on detecting a doubling of the UK community prevalence of lifetime suicide attempt (6·5%) in young adult samples. |
| Quantitative variables | 11 | **Explain how quantitative variables were handled in the analyses. If applicable, describe which groupings were chosen and why:** Our Methods section defines the list of 3 exposure groups, 4 outcomes, and 8 covariates; and how each was used in the analysis. |
| Statistical methods | 12 | **(*a*) Describe all statistical methods, including those used to control for confounding:** We describe our use of multivariable linear and ordinal regression, including justification of the 8 covariates used in the adjusted models. |
|  |  | **(*b*) Describe any methods used to examine subgroups and interactions:** We describe how we tested for an interaction with kinship. |
|  |  | **(*c*) Explain how missing data were addressed:** We explain that levels of missing data were low (<7%) and describe how we used best and worst case scenarios to impute missing values as part of our sensitivity analyses. |
|  |  | **(*d*) If applicable, describe analytical methods taking account of sampling strategy:** We describe our use of a cluster variable to take into account the potential for clustering of responses within HEIs. |
|  |  | **(*e*) Describe any sensitivity analyses:** We describe sensitivity analyses that assessed the impact of missing data and simulated more stringent inclusion criteria for the sampling strategy. |
| Results | | |
| Participants | 13* | **(a) Report numbers of individuals at each stage of study—eg numbers potentially eligible, examined for eligibility, confirmed eligible, included in the study, completing follow-up, and analysed:** We specify numbers of those participating, consenting, and eligible, and present the participant flow in Figure 1. |
|  |  | **(b) Give reasons for non-participation at each stage:** numbers not consenting, not eligible, not indicating exposure group, and not providing at least 1 outcome measure presented in Figure 1. |
|  |  | **(c) Consider use of a flow diagram**: see Figure 1 |
| Descriptive data | 14* | **(a) Give characteristics of study participants (eg demographic, clinical, social) and information on exposures and potential confounders:** Table 1 and text indicates descriptive characteristics by exposure group. |
|  |  | **(b) Indicate number of participants with missing data for each variable of interest**: Table 1 provides proportion of missing values for each covariate of interest by exposure group. |
| Outcome data | 15* | **Report numbers of outcome events or summary measures:** Table 2 presents prevalence (or mean score) for each outcome by exposure group. |
| Main results | 16 | **(*a*) Give unadjusted estimates and, if applicable, confounder-adjusted estimates and their precision (eg, 95% confidence interval). Make clear which confounders were adjusted for and why they were included:** Text and Tables 3 and 4 provide unadjusted and adjusted estimates, with 95% confidence intervals and p-values. |
|  |  | **(*b*) Report category boundaries when continuous variables were categorized**: standard deviation, range, and inter-quartile range reported as appropriate. |
|  |  | **(*c*) If relevant, consider translating estimates of relative risk into absolute risk for a meaningful time period:** N/A |
| Other analyses | 17 | **Report other analyses done—eg analyses of subgroups and interactions, and sensitivity analyses:** We report stratum-specific analyses for relatives and non-relatives of the deceased, following interaction tests. |
| Discussion | | |
| Key results | 18 | **Summarise key results with reference to study objectives:** The start of our discussion summarises the principle findings in relation to our main hypothesis. |
| Limitations | 19 | **Discuss limitations of the study, taking into account sources of potential bias or imprecision. Discuss both direction and magnitude of any potential bias:** Our discussion summarises both the strengths and weaknesses of this study, both in comparison with other potential approaches, and other previously-used approaches. We consider the possibility of either over- or under-estimation of risks given specific potential biases. |
| Interpretation | 20 | **Give a cautious overall interpretation of results considering objectives, limitations, multiplicity of analyses, results from similar studies, and other relevant evidence:** Our discussion sums up the existing literature and comments on the degree to which our findings are consistent with this, and the extent to which they contribute to our understanding of the stigma associated with suicide bereavement. |
| Generalisability | 21 | **Discuss the generalisability (external validity) of the study results:** We explore the degree to which a UK HEI population is generalizable to the rest of the population, either in the UK or internationally. |
| Other information | | |
| Funding | 22 | **Give the source of funding and the role of the funders for the present study and, if applicable, for the original study on which the present article is based:** Our footnotes identify the MRC as the funder, and the limits of their role in this study. |

*Give information separately for exposed and unexposed groups.

**Note:** An Explanation and Elaboration article discusses each checklist item and gives methodological background and published examples of transparent reporting. The STROBE checklist is best used in conjunction with this article (freely available on the Web sites of PLoS Medicine at http://www.plosmedicine.org/, Annals of Internal Medicine at http://www.annals.org/, and Epidemiology at http://www.epidem.com/). Information on the STROBE Initiative is available at www.strobe-statement.org.
